# Shared Control of Supernumerary Robotic Limbs Using Mixed Realityand Mouth-and-Tongue Interfaces

**DOI:** 10.3390/bios15020070

**Published:** 2025-01-23

**Authors:** Hongwei Jing, Sikai Zhao, Tianjiao Zheng, Lele Li, Qinghua Zhang, Kerui Sun, Jie Zhao, Yanhe Zhu

**Affiliations:** State Key Laboratory of Robotics and System, Harbin Institute of Technology, Harbin 150006, China; 19b908070@stu.hit.edu.cn (H.J.); zhengtj@hit.edu.cn (T.Z.); 23b908056@stu.hit.edu.cn (L.L.); 19b908069@stu.hit.edu.cn (Q.Z.); 21b908043@stu.hit.edu.cn (K.S.); jzhao@hit.edu.cn (J.Z.); yhzhu@hit.edu.cn (Y.Z.)

**Keywords:** human–robot interaction, mouth-and-tongue device, human augmentation, wearable robotic limbs, supernumerary robotic limbs

## Abstract

Supernumerary Robotic Limbs (SRLs) are designed to collaborate with the wearer, enhancing operational capabilities. When human limbs are occupied with primary tasks, controlling SRLs flexibly and naturally becomes a challenge. Existing methods such as electromyography (EMG) control and redundant limb control partially address SRL control issues. However, they still face limitations like restricted degrees of freedom and complex data requirements, which hinder their applicability in real-world scenarios. Additionally, fully autonomous control methods, while efficient, often lack the flexibility needed for complex tasks, as they do not allow for real-time user adjustments. In contrast, shared control combines machine autonomy with human input, enabling finer control and more intuitive task completion. Building on our previous work with the mouth-and-tongue interface, this paper integrates a mixed reality (MR) device to form an interactive system that enables shared control of the SRL. The system allows users to dynamically switch between voluntary and autonomous control, providing both flexibility and efficiency. A random forest model classifies 14 distinct tongue and mouth operations, mapping them to six-degree-of-freedom SRL control. In comparative experiments involving ten healthy subjects performing assembly tasks under three control modes (shared control, autonomous control, and voluntary control), shared control demonstrates a balance between machine autonomy and human input. While autonomous control offers higher task efficiency, shared control achieves greater task success rates and improves user experience by combining the advantages of both autonomous operation and voluntary control. This study validates the feasibility of shared control and highlights its advantages in providing flexible switching between autonomy and user intervention, offering new insights into SRL control.

## 1. Introduction

The integration of Supernumerary Robotic Limbs (SRLs) and control systems has significantly enhanced human capabilities in performing complex tasks and operating in dynamic environments [1,2]. Researchers at MIT, Federico Patrietti and H. Asada, introduced Supernumerary Robotic Limbs (SRL) to augment human physical abilities by providing additional robotic limbs that assist in collaborative drilling [3] and ceiling panel installation [4]. Subsequent developments have produced robotic fingers for assistive living operations [5,6,7], limbs for underwater swimming assistance [8], and energy-harvesting flexible limbs [9]. These robotic limbs act as intelligent extensions of the human body, enhancing task execution efficiency and precision.

A critical challenge in SRL control is acquiring task and environmental information to achieve effective human–robot interaction. Traditional control methods utilize various physiological signals, such as movements of redundant limbs [10,11,12], gaze direction [13], electromyography (EMG) [14,15], and electroencephalography (EEG) [16,17]. However, these methods often involve complex data processing or have limited degrees of freedom in control. To address these issues, some studies have shifted to control methods that incorporate voluntary human movement, including voluntary-reactive control for assisting hemiplegic patients in eating [18], voluntary redundant hybrid control for door opening tasks [19], and peg-in-hole assembly tasks [20]. While these methods have alleviated some limitations in the degrees of freedom for redundant limb control, they still face challenges related to operational capability and task constraints, limiting their applicability in real-world scenarios.

In previous studies, mixed reality devices have shown the potential to improve the control and functionality of SRLs [21,22]. These devices offer an immersive interface by overlaying digital information onto the physical world, enhancing spatial awareness and interaction. Vision-based control and feedback provide users with an enriched interaction experience. However, this method has limitations regarding the operational precision of the SRL. When the SRL’s autonomous movements do not meet user needs, it is necessary for users to fine-tune the SRL via certain interfaces. This paper presents a system that integrates a mixed reality device with a mouth-and-tongue interface to achieve shared control of the SRL. Shared control, in this context, refers to the combination of user voluntary control and SRL autonomous control. Existing control methods for SRLs have explored a range of strategies that incorporate voluntary human movements [18,19,20]. These methods reduce the burden of redundant limb control by utilizing human body motion to supplement SRL functions.

Compared to these existing methods, the primary advantage of our approach lies in two key areas: First, the tongue allows for finer and more flexibility movements compared to larger body motions. Second, the system in this paper offers the ability to switch between voluntary and autonomous control dynamically, rather than relying on fixed degrees-of-freedom settings, and it provides rich interactive feedback through the mixed reality interface. However, the system also has certain limitations. While tongue movements are flexible, they may not be as intuitive or convenient as physical body motions for some users. Moreover, the complexity of the system may add to the learning cost for users, resulting in a higher cognitive load.

In our previous research, the mouth-and-tongue interface was preliminarily validated for controlling the SRL, achieving three-degrees-of-freedom control [23]. In this study, this capability is extended by classifying 14 types of mouth-and-tongue interface operations using a random forest method, mapping these to six degrees of freedom in SRL motion and control mode switching. These classifications, combined with multimodal information from the mixed reality device, enable seamless shared control. The main contributions of this paper are as follows:

(1) A shared control method for Supernumerary Robotic Limbs is proposed, achieving multi-degree-of-freedom manipulation through the integration of a mixed reality device and a mouth-and-tongue interface.
(2) Shared control has been successfully applied to assembly tasks, with comparative experiments demonstrating its advantages by combining the efficiency of autonomous operation with the flexibility of user control adjustments.

The remainder of this paper is organized as follows: Section 2 introduces the components of the shared control system, including the mouth-and-tongue control system and the mixed reality interaction interface, which are essential for enabling shared control of the SRL. Section 3 presents the shared control method, explaining the integration of user input and autonomous control, as well as how the system dynamically switches between these modes. Section 4 presents the experimental setup and results, analyzing the system’s performance through the assembly task design and the metrics used to evaluate its effectiveness. Section 5 discusses the experimental results, emphasizing the strengths of the method, limitations, and potential improvements. Finally, Section 6 concludes the paper by summarizing the key findings and the feasibility of the shared control system.

## 2. System Overview

**Figure 1:**
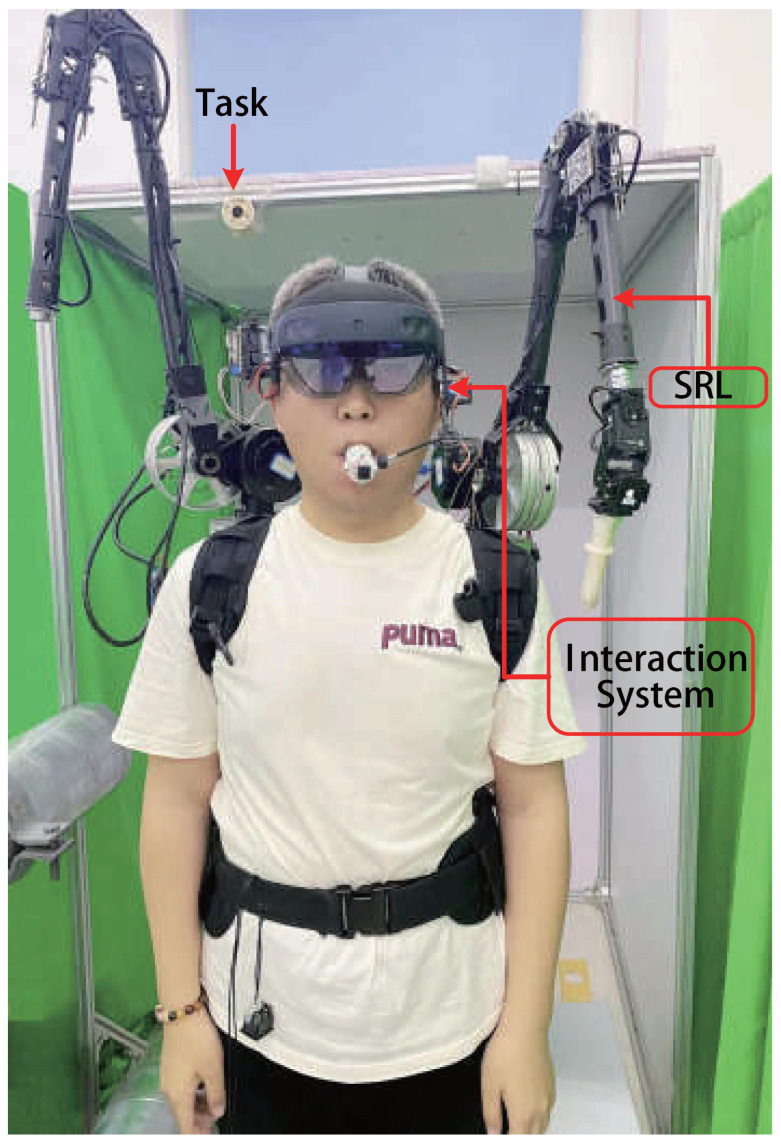
System configuration overview.

The shared control of the SRL is achieved through a combination of user-controlled operations via a mouth-and-tongue interface and automated control facilitated by a mixed-reality device. The entire system, as illustrated in Figure 1, includes the SRL and interaction system.

**Figure 2:**
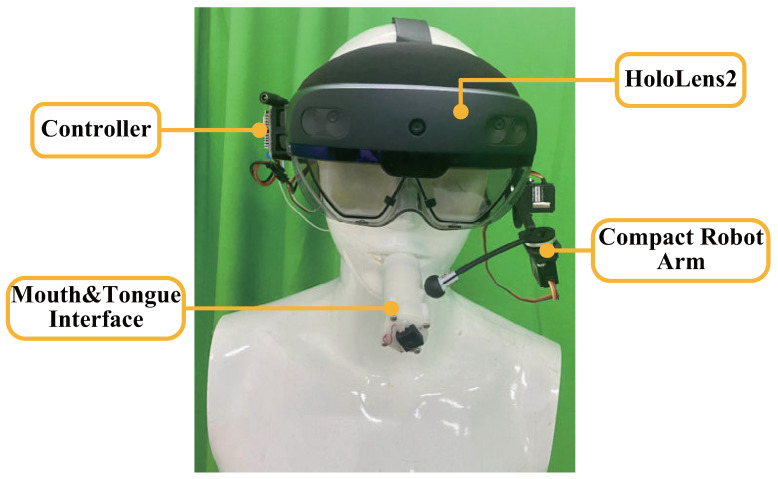
Schematic of the interaction system components.

The interaction system comprises a mixed-reality device and the mouth-and-tongue interface, as shown in Figure 2. The SRL prototype used in this study, which was introduced in our previous work, features a six-degree-of-freedom wearable robotic arm [24]. Although the SRL prototype is a dual-arm robot, only one arm is utilized in this study. The total weight of the system is 9.82 kg, with the SRL system accounting for 9.1 kg, the mixed reality device 0.56 kg, and the mouth-and-tongue interface 0.16 kg.

### 2.1. Mouth-and-Tongue Control System

The mouth-and-tongue control system is a crucial component of our shared control approach, consisting of a compact mouth-and-tongue interface and an electromechanical wearable device. The system aims to provide the user with a voluntary control interface while enabling seamless switching with the autonomous control of the SRL.

In our previous work, we developed an innovative mouth-and-tongue interaction device, which was initially validated for its capability to control the SRL using mouth-and-tongue movements [23]. The device employs Hall sensors and an air pressure sensor to detect the tongue’s manipulation of a control stick and the mouth inhalation and exhalation actions, respectively. This interface allowed users to exert direct control over the SRL through intuitive and natural mouth-and-tongue movements.

Building upon the initial design, we have enhanced the system by incorporating an electromechanical wearable device, as shown in Figure 2. This enhancement addresses the need for more robust and user-friendly control mechanisms. The electromechanical wearable device is a compact robotic arm system that includes a controller, two active rocker arms, and a passive ball joint mechanism, forming a system with two active degrees of freedom and three passive degrees of freedom. This configuration ensures that the device can accommodate a wide range of movements and tasks. Moreover, the electromechanical wearable device facilitates the automatic donning and doffing of the mouth-and-tongue interface, significantly enhancing user convenience and efficiency.

The controller of electromechanical wearable device communicates with the SRL’s main controller via Bluetooth, ensuring a seamless and real-time exchange of control signals. The mouth-and-tongue control system acts as the primary control interface, capturing the user’s voluntary operations and translating them into commands for the SRLs.

### 2.2. Mixed Reality Interaction Interface

We utilize Microsoft HoloLens 2 [25] as the mixed reality device. HoloLens 2 not only provides visual presentations to the user, but also collects various user data through its sensors, including head movement information, gesture recognition based on visual input, gaze tracking, spatial awareness, and voice commands. It also offers audio feedback, allowing researchers to integrate user data with visual and auditory feedback to create a rich interactive interface.

In this study, the HoloLens serves not only as a multimodal interaction interface for the user, but also as a tool for collecting target information that the SRL utilizes for autonomous operation. HoloLens listens to the user’s voice input to trigger the automatic donning of the mouth-and-tongue interface. To assist the user in conveying their intent while using the interface, HoloLens tracks the user’s gaze and records the collision time with the virtual visual interface, triggering the release of the mouth-and-tongue interface when needed.

Communication between the HoloLens 2 and the SRL’s main controller is achieved via a wireless network, enabling real-time data transmission to ensure that the electromechanical wearable system promptly executes user commands. During task execution, HoloLens utilizes Vuforia to recognize assembly hole positions and overlays AR guidance information onto the user’s field of view, providing real-time operational prompts. The SRL can then use the identified assembly hole position information to perform autonomous actions. The combined visual and auditory feedback from HoloLens helps reduce the user’s operational burden and enhances the overall efficiency of the system.

## 3. Shared Control

The shared control integrates user voluntary control based on the mouth-and-tongue interface with robotic autonomous control. In this study, a random forest model is used to recognize the operations of the mouth-and-tongue interface, achieving six degrees of freedom control of the SRL. For practical assembly tasks, a shared control strategy is proposed by combining data from the HoloLens 2 with the mouth-and-tongue operation patterns.

### 3.1. Selection of Mouth-and-Tongue Operations

**Figure 3:**
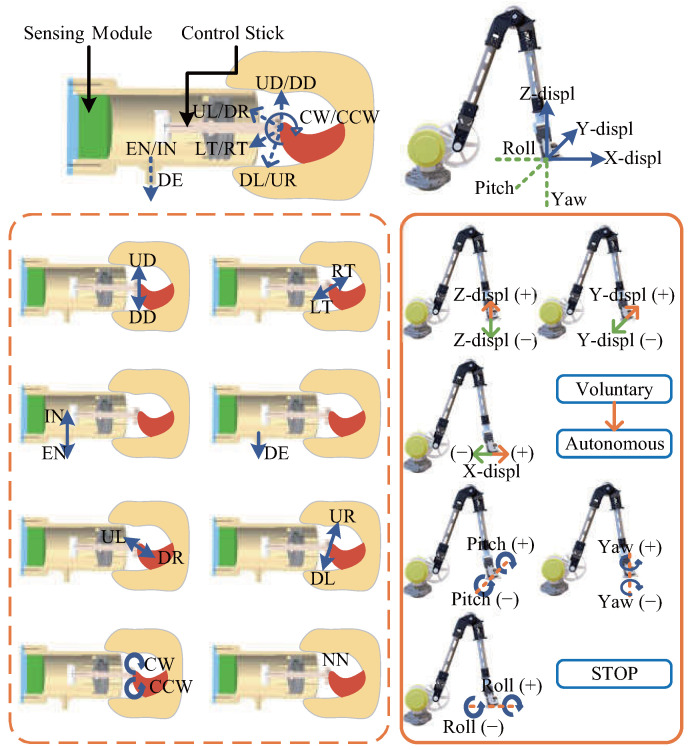
Mapping of mouth-and-tongue interface operations to SRL movements. The operations of the mouth-and-tongue interface are mapped to the six degrees of freedom movements of the SRL’s end effector and the mode switching function.

The control stick of the mouth-and-tongue interface is designed for users to adapt to SRL control. To intuitively map the user’s mouth-and-tongue operations to the voluntary control of the SRL, specific mouth-and-tongue operations are selected, as illustrated in Figure 3. The upward (UD) and downward (DD) movements of the tongue on the device’s control stick are mapped to Z-displ (+) and Z-displ (−) of the SRL along the Z-axis, respectively. Similarly, the left (LT) and right (RT) movements of the tongue on the control stick are mapped to Y-displ (+) and Y-displ (−) of the SRL along the Y-axis. Forward and backward pushes of the tongue on the control stick are difficult to measure, so mouth exhalation (EN) and inhalation (IN) actions are used instead. These actions are mapped to X-displ (+) and X-displ (−) of the SRL along the X-axis.

To fully utilize the tongue’s capability and spatial orientation, downward-right (DR) and upward-left (UL) movements of the tongue on the control stick are mapped to the pitch (+) and pitch (−) of the SRL. Downward-left (DL) and upward-right (UR) movements are mapped to the yaw (+) and yaw (−) of the SRL. Following the human cognitive understanding of spiral movements, clockwise (CW) and counterclockwise (CCW) movements of the tongue on the control stick are mapped to the roll (+) and roll (−) of the SRL.

Additionally, double consecutive exhalations (DE) are categorized as one operation, intended to be mapped as the intention to switch between voluntary and autonomous control modes. Lastly, a no-operation state (NN) of the mouth-and-tongue interface is mapped to stop the SRL’s movement. In total, 14 distinct states of mouth-and-tongue operations are defined to meet the user’s six-degrees-of-freedom control requirements for the SRL, ensuring comprehensive and intuitive control.

### 3.2. Recognition of Mouth-and-Tongue Operations

To map the mouth-and-tongue interface operations to SRL movements, a random forest model is utilized for data training and classification. The random forest model consists of multiple decision trees, each trained on a random subset of the training data and using a random subset of features for node splitting. This approach helps reduce overfitting and improves generalization capabilities. Predictions are made by aggregating the majority votes from all individual decision trees in the random forest,

(1) $$H\left(x\right)=\mathrm{mode}\{h_{1}\left(x\right),\;h_{2}\left(x\right),\ldots ,\;h_{t}\left(x\right)\}$$

where H(x) is the final prediction and $h_{t}\left(x\right)$ is the prediction from the *t*-th decision tree.

The mouth-and-tongue interface collects user control stick operations and breathing actions through four Hall sensors and one air pressure sensor. The data transmission cycle for the interface sensors is 100 ms. Considering feature extraction and control performance, sensor data points are grouped into a training set of 20 points (2 s) for training. Initially, the raw sensor data are preprocessed to remove noise and outliers. The data matrix is denoted as *X*, with dimensions n×m representing *n* samples and *m* sensor data sources.

(2) $$X=\left(\begin{array}{cccc} x_{11} & x_{12} & \cdots & x_{1m}\\ x_{21} & x_{22} & \cdots & x_{2m}\\ \vdots & \vdots & \ddots & \vdots \\ x_{n1} & x_{n2} & \cdots & x_{nm} \end{array}\right)$$

Data cleaning functions eliminate missing and outlier values and a median filter is applied for denoising. Each column of sensor data undergoes median filtering to remove noise.

(3) $$X_{\mathrm{clean}}=\left\{x_{i}|x_{i}\in X\;\mathrm{and}\;\forall j,x_{ij}\neq \mathrm{NaN}\right\}$$

(4) $$X_{\mathrm{denoised}}\left[:,j\right]=\mathrm{medfilt}\left(X_{\mathrm{clean}}\left[:,j\right],\;\mathrm{kernel\_size}=3\right)$$

where medfilt is the median filter function and kernel_size=3 is the window size.

Statistical and time-series features are extracted from the data, including mean, standard deviation, and downward slope. Several statistical features are calculated for each sensor.

(5) $$\mu_{j}=\frac{1}{n}\sum_{i=1}^{n}x_{ij}$$

where $\mu_{j}$ is the mean value of the *j*-th sensor.

(6) $$\sigma_{j}=\sqrt{\frac{1}{n}\sum_{i=1}^{n}{\left(x_{ij}-\mu_{j}\right)}^{2}}$$

where $\sigma_{j}$ is the standard deviation of the *j*-th sensor.

(7) $${\mathrm{slope}}_{j}=\min_{j}\left(\frac{x_{ij+1}-x_{ij}}{t_{j+1}-t_{j}}\right)$$

where ${\mathrm{slope}}_{j}$ represents the downward slope of the *j*-th sensor.

Each sensor includes two statistical features and one time-series feature, resulting in a total of 15 features for the five sensors, which are then standardized. To optimize the performance of the random forest classifier, randomized search is used to search for the best combination of hyperparameters. The hyperparameter range, including the number of decision trees, the maximum depth of the trees, the minimum number of samples required to split an internal node, the minimum number of samples required to be at a leaf node, and the number of features to consider when looking for the best split are defined.

A total of 100 iterations were chosen to perform the random search across the hyperparameter space, and a 5-fold cross-validation was used to evaluate the performance of each parameter combination. The optimal set of hyperparameters is then determined and applied to the subsequent model training process.

### 3.3. Shared Control Strategy

Shared control aims to achieve a natural switch between user voluntary control and SRL autonomous control. This study constructs a shared control strategy using a common assembly task for SRL applications as an example. The mouth-and-tongue interface operations are defined in 14 modes, mapped to the six degrees of freedom control and mode switching of the SRL. equipment, while the SRL assists with pin-hole assembly. After the SRL autonomously performs coarse alignment of the pin and hole, the user fine-tunes the assembly through voluntary control. The SRL’s autonomous control of pin-hole alignment helps reduce the user’s workload and increase efficiency.

**Figure 4:**
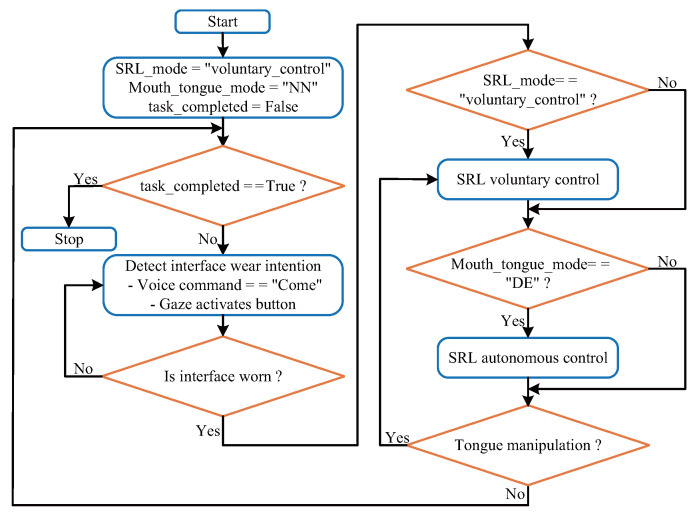
Flowchart of Shared Control Strategy.

The flowchart, as shown in Figure 4, illustrates the shared control strategy for the SRL, detailing the interaction between voluntary and autonomous control modes and the process of attaching or detaching the interface. HoloLens facilitates interaction between the user and SRL using voice, gaze, and visual information. The specific shared control strategy is illustrated in Algorithm 1. To avoid interfering with the user’s handwork, a voice command (“Come”) triggers the mouth-and-tongue interface’s electric-wearing device. A virtual button activated by gaze is set to detach the mouth-and-tongue interface. Initially, SRL operates in voluntary control mode. HoloLens recognizes environmental information and aligns the assembly scene with the real-world scene, providing task operation prompts and installation hole information to the user. Using the tracking method proposed in our previous research [22], synchronization between virtual scene coordinates and the real-world scene is achieved.
**Algorithm 1** Shared control strategy for SRL with mouth-and-tongue interfaceINITIAL VALUES SRL_mode←“voluntary_control”, Mouth_tongue_mode←“NN”, task_completed←False**while** not task_completed **do**   **while** not mouth_tongue_interface_attached **do**       **if** voice_command == “Come” **then**           attach_mouth_tongue_interface()       **end if**   **end while**   **if** gaze_activates_virtual_button() **then**       detach_mouth_tongue_interface()   **end if**   **if** SRL_mode==“voluntary_control” **then**       align_scene_with_real_world(HoloLens)       SRL_voluntary_control()   **end if**   **if** Mouth_tongue_mode==“DE” **then**       SRL_mode←“autonomous_control”   **end if**   **if** SRL_mode==“autonomous_control” **then**       align_end_effector_with_installation_hole(gaze_position)   **end if**   **if** Mouth_tongue_mode≠“DE” **and**   Mouth_tongue_mode≠“NN”
**then**       SRL_mode←“voluntary_control”   **end if**   **if** installation_task_completed() **then**       task_completed←True   **end if****end while**

Subsequently, the user switches the mode to autonomous control by performing double consecutive exhalations (DE). HoloLens identifies the hole and provides visual overlays to the user. The user gaze is used to confirm the intent to target the hole. SRL then aligns the pin on the end-effector with the hole. To avoid collisions, a positional offset is set, allowing the pin to stop at a certain distance along the axis of the hole. When the user performs other operations on the tongue interface, the mode automatically switches back to voluntary control. The user adjusts the position of the pin and fine-tunes its posture to complete the installation task.

The voluntary control of SRL is based on the classification of operations of the mouth and tongue. Each operation classification *C* has been mapped to the six degrees-of-freedom (DOFs) motion of SRL, denoted as M, which includes three translational DOFs T and three rotational DOFs R.

The speed is V, which increases linearly with the number of times *N* the classification $C_{i}$ is entered continuously and stops increasing after reaching a certain threshold $N_{\max}$. The speed control formula is expressed as

(8) $$V_{i}=\min\left(\frac{N_{i}}{N_{\max}}V_{\max},V_{\max}\right)$$

where $V_{\max}$ is the maximum speed.

Autonomous control aligns the SRL with the target hole along the *x*-axis while maintaining a positional offset Δx. Let P and $P_{\mathrm{target}}$ represent the position vectors of SRL and the target hole, respectively. The position error is given by

(9) $$E_{\mathrm{pos}}=\left(P_{\mathrm{target}}+\Delta x\right)-P$$

The control of adjusting the position based on error feedback can be expressed as

(10) $$P\left(t+1\right)=P\left(t\right)+K_{p}E_{\mathrm{pos}}\left(t\right)$$

where $K_{p}$ is the proportional gain matrix for position control and *t* is the time step.

For attitude control, let A and $A_{\mathrm{target}}$ denote the attitude matrices of SRL and the target, respectively. The attitude error is

(11) $$E_{\mathrm{att}}=A_{\mathrm{target}}-A$$

The control of adjusting the attitude based on error feedback can be expressed as

(12) $$A\left(t+1\right)=A\left(t\right)+K_{a}E_{\mathrm{att}}\left(t\right)$$

where $K_{a}$ is the proportional gain matrix for attitude control and *t* is the time step.

## 4. Experiment

### 4.1. Training Data Acquisition

The data collection was approved and supervised by the Medical Ethics Committee of Harbin Institute of Technology (Approval No. HIT-2024039). Ten healthy subjects (aged 26–29 years; weight 75–85 kg; height 1.70–1.78 m) participated in the experiment and familiarized themselves with the experimental procedures approximately 0.5 h before the experiment. A standardized data collection interface was designed, displaying raw data from the mouth-and-tongue interface and providing operational instructions to the subjects. Five were involved in the collection of training data. Each subject performed the experiment twice and was required to follow the interface prompts to execute 14 different operations in sequence. Each operation was repeated five times with a 2 s interval between each repetition. After completing the data collection for all subjects, a total of 700 training datasets were obtained for subsequent model training.

### 4.2. Recognition of Mouth-and-Tongue Operations

The collected dataset was divided into a training set with 560 samples and a test set with 140 samples. A random forest classifier [26], optimized using randomized search, was employed to classify the sensor data. The optimal hyperparameters determined are the number of decision trees: 100; maximum depth: 5; minimum samples split: 15; and minimum samples leaf: 10. The classifier exhibited accuracy on both the training and test datasets, achieving 98.04% and 98.57%, respectively. These accuracy values indicate that the model generalizes well to unseen data.

**Figure 5:**
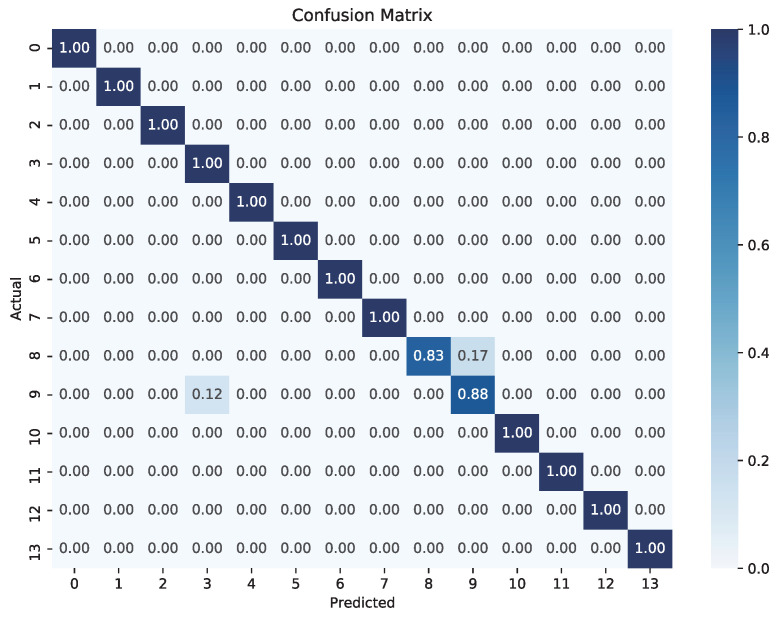
Confusion matrix representing classification accuracy.

The confusion matrix for the mouth-and-tongue operation recognition results of the five subjects is shown in Figure 5. It can be observed that most categories are predicted very accurately, with only a few misclassifications. For instance, class 8 is misclassified as class 9, and class 9 is misclassified as class 3. This misclassification could be due to the complexity and similarity of the tongue’s clockwise and counterclockwise movements when controlling the joystick.

**Figure 6:**
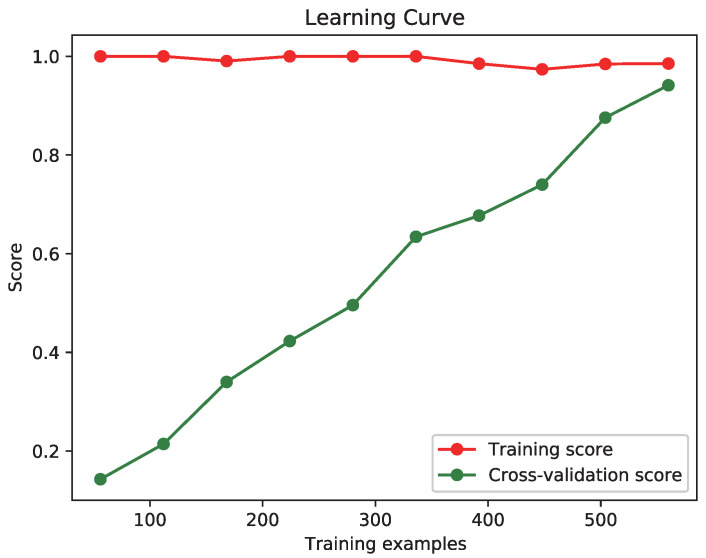
Learning curve demonstrating model performance.

In this study, 5-fold cross-validation is applied to evaluate the robustness and generalizability of the machine learning model. The data collected from all subjects is combined and randomly divided into five equal parts. During each iteration, four parts are used for model training, while the remaining part is used for testing. This process is repeated five times, ensuring that each part serves as the test set once. This method ensures that the algorithm’s performance is robust and not overly dependent on any single subject’s data. The model’s learning curve is depicted in Figure 6, which shows the scores for the training set and the cross-validation set as the number of training samples increased (from 10% to 100%). The model’s average cross-validation accuracy was 94.10%, indicating that the model has good generalization ability and can achieve relatively stable performance on new datasets.

### 4.3. Assembly Task Experiments

**Figure 7:**
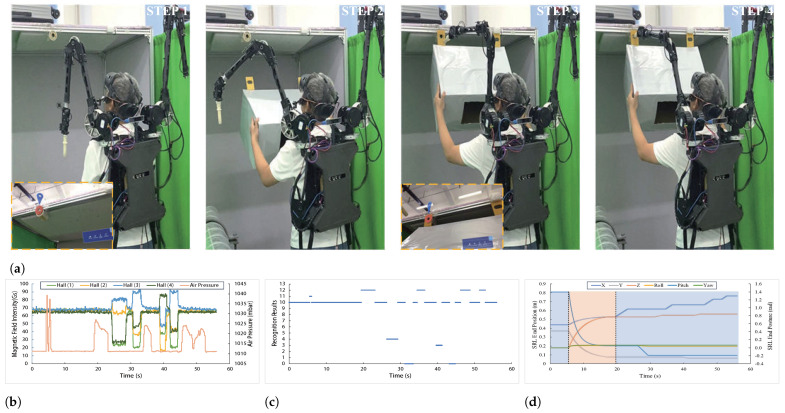
Performance of shared control in assembly tasks. (**a**) The step-by-step assembly task demonstration using shared control of SRL. The yellow dashed box represents the HoloLens interface. (**b**) Raw data from the mouth-and-tongue interface during assembly. (**c**) Classification results of mouth-and-tongue interface operations using the Random Forest model. Categories 0 to 13 correspond to UD, DD, LT, RT, UL, DR, DL, UR, CCW, CW, NN, DE, EN, and IN. (**d**) The curve of the SRL’s reference end-effector position and orientation, where the blue shaded area represents the voluntary control phase, and the pink area represents the autonomous control phase.

To evaluate the proposed shared control method, ten subjects are recruited to perform real assembly tasks. Each subject is equipped with the SRL and the shared interactive system, as shown in Figure 7a. This experimental design includes three comparative experiments (shared control, autonomous control, and voluntary control), with each subject completing five trials in one experiment and a 10 min rest period between each trial. During the experiment, subjects are guided to stand in different positions to increase the diversity of experimental conditions, thereby enabling a more comprehensive evaluation of the effectiveness of the control methods.

In the shared control trials, subjects use autonomous control for coarse positioning and complete fine adjustments through voluntary control. In the voluntary control trials, subjects rely solely on the tongue interface to control the 6-DOF movement of the SRL end-effector to complete the assembly task. In the autonomous control trials, subjects rely on the Hololens to detect the hole position, after which the SRL executes a pre-programmed insertion action. The results primarily showcase the experimental process of shared control, raw sensor data, classification results, and SRL reference trajectory data. Additionally, the three control conditions (shared control, autonomous control, and voluntary control) are compared regarding task completion time and user experience to assess the effectiveness of the different control methods.

During the shared control experiments, the task follows a structured process facilitated by the SRL and interactive system. Step 1 involves target recognition, where the HoloLens identifies the position of the installation hole and highlights it within the subject’s field of view. The HoloLens provides installation prompts to guide the user through the task. Step 2 sees the SRL entering the task initialization state, preparing to begin the assembly task. Following this, Step 3 involves the SRL autonomously aligning its end-effector with the installation hole. However, due to factors such as visual recognition inaccuracies or subject movements, slight misalignments occur. Step 4 allows subjects to switch to voluntary control at any time, where they can fine-tune the SRL’s position via the mouth-and-tongue interface to complete the installation task. To enhance safety, subjects confirm their intent to proceed with the installation by focusing their gaze on the hole and performing a double exhalation. This structured sequence combines autonomous control for coarse positioning and user intervention for precise adjustments, facilitating an efficient and flexible approach to completing the assembly task.

The raw mouth-and-tongue interface data and recognition results during experiments are shown in Figure 7b,c. The sensor data in Figure 7b corresponds to the operational actions in experiments, such as a continuous increase in air pressure indicating two blowing actions, and changes in Hall sensors reflecting tongue movements. By comparing operational actions reflected by sensor data with the model’s classification outputs, the performance of model recognition can be observed. There is some lag, which may be due to the sensor data refresh rate and model computation speed. Additionally, reference data of SRL are recorded, as shown in Figure 7d. Around 5 s, subjects switch SRL to autonomous control by performing double consecutive exhalations (DE). Position and orientation of SRL smoothly transition to align with the hole. Around 20 s, subjects switch to voluntary control by pushing the joystick upwards (UD), continuing until the task is completed. SRL’s trajectories are generally smooth throughout the process, demonstrating basic control capabilities of shared control system.

**Figure 8:**
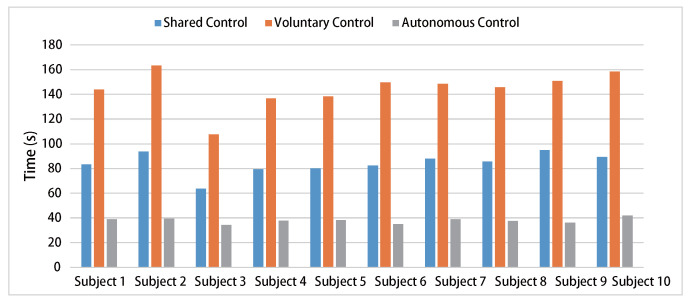
Time statistics of task completion by subjects under three control methods.

A statistical analysis is conducted on the task success rate and completion time of the ten subjects under three experimental conditions. The success rates for both shared control and voluntary control are 100%, while the success rate for autonomous control is only 48%. Regarding task completion time, the ranking from longest to shortest is voluntary control, shared control, and autonomous control, as shown in Figure 8. Although autonomous control demonstrates an advantage in task completion efficiency, its success rate is low due to the lack of user adjustments. Therefore, shared control offers a relatively balanced advantage in both task efficiency and success rate. It combines the benefits of autonomous operation with the flexibility of user intervention.

**Figure 9:**
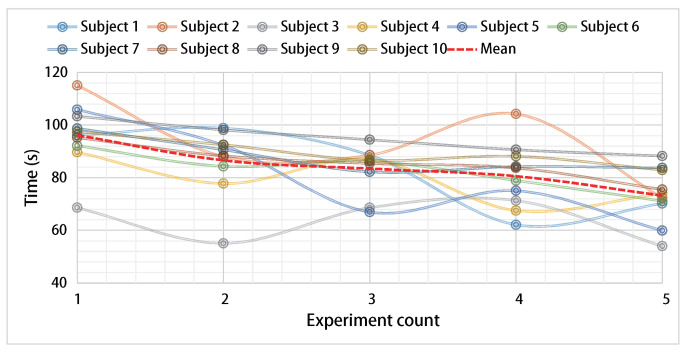
Variation in task completion time across trials for subjects using shared control.

Figure 9 illustrates the relationship between task completion time and the number of operations for the ten subjects using shared control. It shows that as the number of operations increases, the subjects’ efficiency improves. This indicates that training can further enhance the efficiency of task completion through shared control.

The results of the user feedback survey from the subjects are shown in Table 1. The user feedback results show that the shared control method outperforms both direct voluntary control and autonomous control. It receives higher scores in control effectiveness (4.4), ease of task completion (4.6), and overall operation experience (4.5). Although all methods are rated similarly for intuitiveness, shared control offers a more efficient and satisfying experience, combining autonomous and voluntary control to reduce user effort while maintaining system responsiveness.

In summary, the interactive system combining mixed reality and the mouth-and-tongue interface successfully achieves shared control of the SRL and is tested in assembly tasks. The comparative experiments demonstrated the advantages of shared control, which integrates the efficiency of autonomous operation with the flexibility of user-controlled adjustments.

## 5. Discussion

This paper presents an interactive system that integrates a mixed reality device with a mouth-and-tongue interface to enable shared control of the SRL. The system allows users to switch between voluntary and autonomous control, combining the advantages of both to enhance task performance. Experimental results demonstrate that shared control improves operational efficiency and flexibility compared to purely autonomous or voluntary approaches.

However, several challenges remain. A limitation is the delay in operation due to sensor data refresh rates and model computation time. Although users can adapt to this delay, it remains an area for future improvement. Additionally, users need invest time in learning how to operate both the MR device and the mouth-and-tongue interface, which may affect initial usability.

**Future work will focus on enhancing the responsiveness of the system by addressing** these delays and improving the user interface for more intuitive control. Additionally, there is potential to increase the system’s autonomy and expand the range of actions recognized by the mouth-and-tongue interface, which would further enhance the user experience and increase the system’s applicability in more tasks.

## 6. Conclusions

This paper introduces a shared control method that enables users to achieve flexible and multi-degree-of-freedom control of the SRL by seamlessly switching between voluntary and autonomous control. The proposed interactive system integrates a mixed-reality device with a mouth-and-tongue interface, employing a random forest model to classify 14 distinct mouth-and-tongue operations mapped to SRL control. The results of comparative experiments on assembly tasks validate both the accuracy of the classification and the effectiveness of the shared control strategy. While the autonomous mode offers efficiency, shared control achieves a balanced combination of machine autonomy and human input, leading to higher task success rates and an enhanced user experience.This work demonstrates the practical feasibility of shared control, offering new perspectives on SRL control by effectively combining user flexibility with automation.

## Figures and Tables

**Table 1:** User feedback evaluation.

Evaluation Criteria	Autonomous Control	Voluntary Control	Shared Control
Intuitiveness of Operation (1–5)	4.1	4.2	4.2
Control Effectiveness (1–5)	2.5	3.6	4.4
System Response Speed (1–5)	4.2	3.2	4.5
Ease of Task Completion (1–5)	2.8	3.6	4.6
Overall Operation Experience (1–5)	3.0	3.6	4.5

## Data Availability

The data presented in this study are available on request from the corresponding author. The data are not publicly available due to the project being in a confidential phase, and institutional regulations currently prevent sharing the data.

## Abbreviations

The following abbreviations are used in this manuscript:

SRL	Supernumerary Robotic Limb
EMG	Electromyography
EEG	Electroencephalography
MR	Mixed Reality
